# HIV Reservoir Decay and CD4 Recovery Associated With High CD8 Counts in Immune Restored Patients on Long-Term ART

**DOI:** 10.3389/fimmu.2020.01541

**Published:** 2020-07-23

**Authors:** Lu-Xue Zhang, Yan-Mei Jiao, Chao Zhang, Jin-Wen Song, Xing Fan, Ruo-Nan Xu, Hui-Huang Huang, Ji-Yuan Zhang, Li-Feng Wang, Chun-Bao Zhou, Lei Jin, Ming Shi, Fu-Sheng Wang

**Affiliations:** ^1^Peking University 302 Clinical Medical School, Beijing, China; ^2^Treatment and Research Center for Infectious Diseases, Fifth Medical Center of Chinese PLA General Hospital, Beijing, China

**Keywords:** HIV, CD8 counts, reservoir decay, CD4 recovery, long-term antiretroviral therapy

## Abstract

**Background:** Whether varying CD8 counts influence the human immunodeficiency virus (HIV) reservoir and CD4 restoration in patients with CD4 counts ≥ 500 cells/μL after long-term antiretroviral therapy (ART) remains unknown. In this study, we analyzed relationships between CD8 levels and viral reservoir decay or CD4 recovery in immune restored patients on long-term ART.

**Methods:** Chronic HIV-infected patients who received 5 years of ART with CD4 counts ≥ 500 cells/μL were grouped according to CD8 counts: CD8 <500 (Group 1), 500–1,000 (Group 2), and ≥1,000 cells/μL (Group 3). CD4 recovery, viral decay, CD8 T-cell function, and their correlations were analyzed during ART among the three groups.

**Results:** Dynamics of viral decay and CD4 recovery were different among the three groups. Both viral decay and CD4 recovery were higher in Group 3 than the other two groups after 5 years of ART, mainly during years 3–5 of ART. Higher expression levels of Ki67 while PD-1 levels were lower on CD8 T-cells in Group 3 compared with the other groups, and Group 3 showed stronger CD8 T-cells functional capacity after 3 years of ART. Reduced HIV DNA levels and increased CD4 counts between years 3 and 5 of ART were positively correlated with CD8 counts and function.

**Conclusions:** High CD8 counts are beneficial for persistent viral decay and CD4 recovery in immune restored patients during long-term ART.

## Introduction

Antiretroviral therapy (ART) has markedly increased CD4 counts in HIV-infected patients and reduced AIDS-related morbidity and mortality. In the past, significantly more attention has been paid to CD4 counts while the CD8 T-cell compartment was relatively underappreciated. CD4 recovery to above 500 cells/μL is frequently observed in the patients under effective ART, but CD8 counts are consistently elevated even after long-term treatment. Elevation of CD8 counts is associated with increased immune anergy and risks of non-AIDS-related clinical events in HIV-infected patients during ART (1, 2).

The viral reservoir size and CD4 counts are two important measurements to determine the effect of ART after plasma viral loads remain at non-detectable levels. Previous studies showed that CD8 counts were positively correlated with the viral reservoir (3–5), and high CD8 counts were associated with a poor increase in CD4 T-cells during ART (1, 6). These results were partly attributed to CD8 T-cell exhaustion and a decrease in functional capacity (7). During HIV infection, poor CD8 T-cell response to control virus may be due to absence of CD4 T-cell help. However, CD8 T-cells play an important role in the control of HIV replication (8) and HIV reservoir (9–11). Previous studies have focused on the impact of different levels of CD8 T-cells on virus and CD4 recovery but did not consider if CD4 counts rebounded to normal levels (1, 4, 6). CD4 recovery to >500 cells/μL is a standard marker for immune restoration (12). For patients with CD4 recovery above 500 cells/μL after long-term ART, the impact of different CD8 levels on viral reservoir decay and CD4 recovery is largely unknown.

In this study, we enrolled patients with immune restoration and subdivided them into three groups according to CD8 counts after 5-years of ART. We observed different patterns of viral decay and CD4 recovery among these three groups. Counter to the tendencies observed during the first year of ART, the CD8 high expressing group demonstrated persistent viral reservoir decay and an additional increase in CD4 counts compared to the other two groups during years 3–5 of ART, and this phenomenon is associated with increased CD8 counts and functional capacity. These findings suggest that high CD8 counts may sustain viral reservoir decay and CD4 recovery in immune restored patients during long-term effective ART.

## Methods

### Study Design and Participant

**Figure 1:**
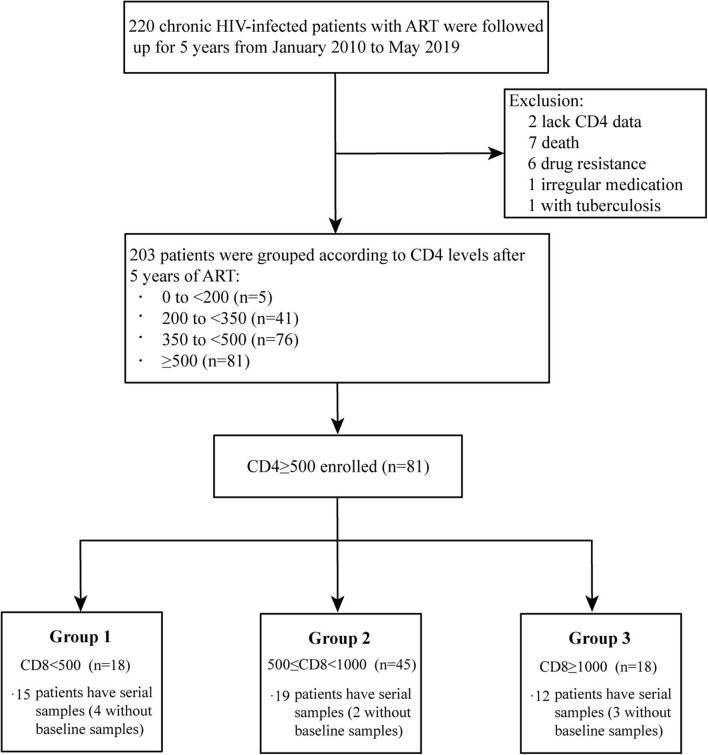
Flow chart of participants enrolled in this study.

HIV/AIDS patients who underwent 5-years of ART during the period of January 2010 to May 2019 in Fifth Medical Center of Chinese PLA General Hospital were enrolled in this study. We excluded patients with missing CD4 data, death, drug resistance, irregular medication, and HIV-related opportunistic infections. The remaining 203 patients were divided into groups according to different CD4 and CD8 levels after 5-years of ART (Supplementary Figure 1). Participants were eligible for our study if they achieved immune restoration (undetectable plasma HIV RNA levels within 6 months of ART and two CD4 counts >500 cells/μL after 5-years of ART). Unlike the uniform standard for CD4 T-cell stratification in HIV/AIDS, there is no uniform standard for the categorization of CD8 T-cells at present. In our study, CD8 counts were stratified based on relevant clinical studies (1, 7, 13, 14). Following these selection criteria, 81 eligible participants were grouped on the basis of three different categories of two CD8 counts after 5-years of ART: 18 in Group 1 (CD8 counts <500 cells/μL, G1), 45 in Group 2 (CD8 counts from 500 to <1000 cells/μL, G2), and 18 in Group 3 (CD8 counts ≥1,000 cells/μL, G3). There were 15 (4 without baseline samples), 19 (2 without baseline samples), and 12 (3 without baseline samples) participants with serial samples in G1, G2, and G3, respectively (Figure 1).

This study was approved by the institutional review boards of Fifth Medical Center of Chinese PLA General Hospital and the study subjects gave informed consent in line with the Declaration of Helsinki.

### Data and Sample Collection

Data were collected as follows: sex, age at ART initiation, route of infection, CD4 counts, CD8 counts, viral load, and ART regiment. At the same time, blood samples were obtained pre-ART (if available), and at years 1, 3, and 5 on treatment. Peripheral blood mononuclear cells (PBMCs) were stored in liquid nitrogen before use. Plasma samples were frozen at −80°C until use.

### Plasma HIV RNA, CD4, and CD8 Counts Determination

CD4 and CD8 counts were determined by flow cytometry (FACS Caliber, BD Biosciences, New Jersey, USA), and HIV RNA load was quantified using the COBAS AmpliPrep/TaqMan real-time RT-PCR Test (Roche, CA, USA).

### HIV DNA Quantification

Extraction of total cellular DNA from thawed PBMCs was measured by QIAamp DNA Mini Kit (Qiagen, Valencia, California). HIV DNA quantitative detection kit (SUPBIO, Guangzhou, China) was used to amplify and quantify the cell-associated HIV DNA. The lower limit of detection was 20 copies/1 × 10^6^ cells, and the quantification range was 50~1 × 10^6^ copies/1 × 10^6^ cells.

### Flow Cytometric Analysis

The following antibodies were used in three panels: CD3 BV421 (clone SK7), CD3 BV510 (clone HIT3a), CD8 APC-H7 (clone SK1), CD4 APC-H7 (clone RPA-T4), CD45RA BV510 (clone HI100), CCR7 PE-Cy7 (clone 3D12), HLA-DR BV421 (clone G46-6), and Ki67 BV421 (clone B56) were obtained from BD Bioscience. CD4 FITC (clone SK3) and programmed death-1 (PD-1) PE (clone EH12.2H7) were obtained from BioLegend (San Diego, CA, USA). CD3 APC (clone UCHT1) and CD38 FITC (clone HIT2) were obtained from QuantoBio (Beijing, China). Cryopreserved PBMCs had more than 85% viability after thawing, then were washed and surface stained for 20 min at room temperature. Cells were then washed and intracellular stained after being permeabilized and fixed using the Permeabilization/Fixation Kit (eBioscience, Waltham, MA, USA).

To evaluate total and HIV-specific CD8 T-cells responses, thawed PBMCs were stimulated with anti-CD3 antibody (clone OKT3, 1 μg/mL, BioLegend) or overlapping peptides comprised by HIV env, gag, and pol (1 μg/mL, respectively, JPT, Berlin, Germany) for 6 h at 37°C in a humidified incubator with 5% CO_2_. The costimulatory antibodies CD28 and CD49d (clone CD28.2 and 9F10, 1 μg/mL, respectively, BioLegend), along with the transport inhibitor Brefeldin A (3 μg/mL, eBioscience) and the CD107a FITC (clone H4A3, BD Bioscience) were added into each stimulus condition. After being stained with surface antibodies (CD4 BV510 [clone SK3, BD Bioscience], CD3 BV510 [clone HIT3a, BD Bioscience], and CD8PerCP [clone HIT8a, QuantoBio]) in two antibody panels, cells were permeabilized and stained with intracellular IFN-γ BV421 (clone B27, BD Bioscience), granzyme-B Alexa Fluor 647 (clone GB11, BD Bioscience), TNF-α PE (clone MAb11, BioLegend), and perforin PE-Cy7 (clone B-D48, BioLegend). Stained cells were fixed in 1% paraformaldehyde and acquired using a BD Canto within 24 h.

### HIV Antibody Measurement

Specific antibody responses were measured with 20 μL of thawed plasma by enzyme immunoassay according to the manufacturer's instructions (Western blot [WB] kit 2.2, MP Diagnostics, Singapore), against 10 HIV viral proteins (gp160, gp120, p66, p55, p51, gp41, p39, p31, p24, and p17). Methods of analyzing the WB strips have been previously published (15). When the gray-scale value of each antigen was ≥50, 10–49, or 0–9% of those calculated in the strong positive control for the same antigen, the corresponding value was assigned a score 1, 0.5, or 0, respectively (Figure 3C, left panel). Finally, a total WB score of each strip was assigned in the sum of the number of positive and weak responses (from 0 to 10).

### Statistical Analyses

Statistical analyses were performed using SPSS 23.0 software (Chicago, IL, USA). The non-parametric Kruskal–Wallis test for continuous variables and the Fisher's exact test for categorical variables were used for multiple comparisons between groups. The Mann–Whitney U test was used for comparison of variables between two groups. The slopes of CD4 counts and HIV DNA over time were estimated by fitting participant-specific linear regressions (16). Correlations were analyzed using the Spearman test. Two-sided *P* < 0.05 was considered statistically significant.

## Results

### Study Participant Characteristics

Baseline characteristics were similar among the three groups, except that participants in G3 had higher pre-ART CD8 counts compared with the other two groups (all *P* < 0.05, Table 1). Similar to that in Table 1, the characteristics of participants with serial samples in the three groups are shown in Supplementary Table 1. Plasma viral loads of all participants had reduced less than the lower limit of detection within 6 months of ART and remained undetectable during the follow-up periods.

**Table 1:** Patient characteristics in the three groups analyzed.

	**Group 1 (*N* = 18)**	**Group 2 (*N* = 45)**	**Group 3 (*N* = 18)**	***P***
Male	16 (88.9)	41 (91.1)	17 (94.4)	1.000
Mode of transmission				
Male-to-male sex	8 (44.4)	30 (66.7)	13 (72.2)	0.168
Heterosexual	7 (38.9)	11 (24.4)	5 (27.8)	0.516
Blood or plasma transfusion	0	1 (2.2)	0	1.000
Undetermined	3 (16.7)	3 (6.7)	0	0.173
Age at ART initiation (years)	43 (37-50)	34 (28-45)	29 (27-45)	0.094
Pre-ART plasma HIV RNA (log_10_ copies/mL)	4.02 (3.33–4.95)	4.03 (3.35–5.35)	4.08 (3.37–5.35)	0.212
Pre-ART CD4 T-cell count (cells/μL)	366 (274–501)	334 (254–482)	351 (241–480)	0.799
Pre-ART CD8 T-cell count (cells/μL)	719 (517–926)^Δ^	927 (706–1325)^▴^	1335 (924–1916)^Δ^^▴^	0.000
ART regimens at initial stage				
2 NRTIs + 1 NNRTIs	18 (100)	44 (60.0)	18 (100)	1.000
2 NRTIs + 1 PIs	0	1 (2.2)	0	1.000

*Data are n (%) or median (IQR). ART, antiretroviral therapy; HIV, human immunodeficiency virus; IQR, interquartile range; NRTIs, nucleoside reverse transcriptase inhibitors; NNRTIs, non-nucleoside reverse transcriptase inhibitors; PIs, protease inhibitor*.

Δ *P < 0.001 (G1 vs. G3)*,

▴ *P = 0.002 (G2 vs. G3)*.

### Longitudinal Changes of CD4 Counts During ART

**Figure 2:**
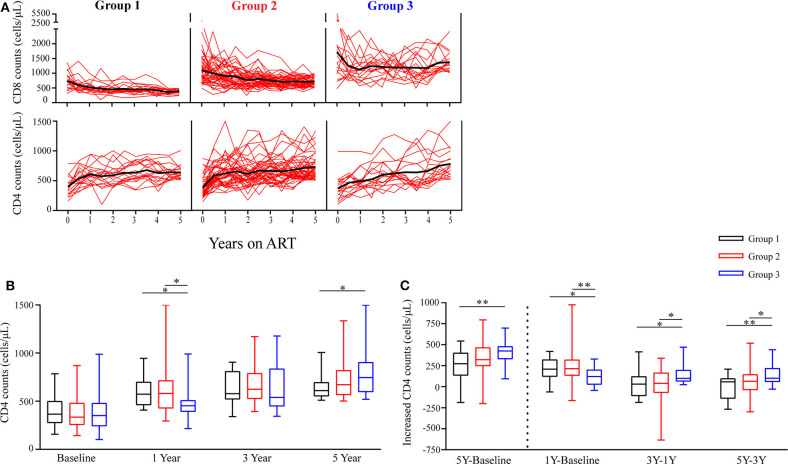
Longitudinal changes in CD4 counts and CD8 counts during ART. **(A)** Time trajectories of CD8 counts and CD4 counts over 5 years in participants receiving ART. Individual trajectories are shown with red lines, mean values are indicated by thick black lines. **(B)** Statistical analysis of CD4 counts in the three groups at baseline, years 1, 3, and 5 on ART. **(C)** Comparisons of increased CD4 counts from three different groups are assessed from baseline to year 5, from baseline to year 1, from years 1 to 3, and from years 3 to 5. Group 1 (G1, *n* = 18 participants), Group 2 (G2, *n* = 45 participants), Group 3 (G3, *n* = 18 participants). In **(B,C)**, boxes show median and IQR, and whiskers are minimum and maximum. *P < 0.05, **P < 0.01.

To investigate changes of circulating CD4 and CD8 counts among the three groups of the participants during ART, we found that almost all participants had a substantial increase in CD4 counts after 5 years of ART (median CD4 counts at baseline, G1 vs. G2 vs. G3, 366 vs. 334 vs. 351 cells/μL; median CD4 counts at year 5 of ART, G1 vs. G2 vs. G3, 611 vs. 672 vs. 746 cells/μL, Figures 2A,B). However, the trajectories of CD4 counts were different among the three groups. The median increased CD4 counts were 209, 214, and 122 cells/μL in G1, G2, and G3 in the first year of ART, respectively; however, the median increased CD4 counts of each group were 58, 64, and 101 cells/μL between years 3 and 5 of ART, respectively (Figure 2C). There was a lowest increase in CD4 counts in G3 within 1 year of ART, while G3 patients had a significant increase of CD4 counts during years 3–5 of ART and had higher CD4 counts after 5 years of ART than the other groups (Figures 2B,C).

Furthermore, Table 2 shows the slopes of CD4 counts during years 0–1, 1–3, and 3–5 of ART, respectively. We found that within 1 year of ART, the slopes of CD4 counts in G3 were significantly lower compared to G1 and G2 (*P* = 0.046, *P* = 0.013); however, the slopes of CD4 counts in G3 were significantly higher compared to G1 and G2 after 3 years of ART (all *P* < 0.05). These results were consistent with above-mentioned results about variation trends of CD4 counts over time among the three groups.

**Table 2:** Slopes of CD4 counts and HIV DNA over time.

**Slope/Year**	**Group 1**	**Group 2**	**Group 3**	***P***
CD4 counts (cells/μL)	*N =* 18	*N =* 45	*N =* 18	
Baseline to year 1 of ART	209 (120, 323)^Δ^	214 (131, 321)^▴^	121 (25, 202)^Δ^^▴^	0.046
Year 1 to Year 3 of ART	16 (−55, 61)^☆^	20 (36, 83)^⋆^	50 (32, 99)^☆^^⋆^	0.039
Year 3 to Year 5 of ART	29 (−61, 49)^°^	32 (−20, 73)^•^	50 (29, 110)^°^^•^	0.012
HIV DNA (log_10_ cps/10^6^PBMC)	*N =* 15	*N =* 19	*N =* 12	
Baseline to year 1 of ART	−0.400 (−0.580, −0.270)^a^	−0.430 (−0.540, −0.250)^b^	−0.320 (−0.520, −0.205)^c^	0.718
Year 1 to Year 3 of ART	−0.025 (−0.060, 0.105)	−0.025 (−0.080, 0.028)	−0.044 (−0.096, 0.010)	0.617
Year 3 to Year 5 of ART	0.035 (−0.010, 0.085)^#^	−0.015 (−0.050, 0.011)	−0.018 (−0.061, 0.014)^#^	0.026

*Data are median (IQR). ART, antiretroviral therapy; cps, copies; HIV, human immunodeficiency virus; IQR, interquartile range*.

a *Data from 11 patients*.

b *Data from 17 patients*.

c *Data from 9 patients*.

Δ *P = 0.046 (G1 vs. G3)*,

▴ *P = 0.013 (G2 vs. G3)*,

☆ *P = 0.031 (G1 vs. G3)*,

⋆ *P = 0.018 (G2 vs. G3)*,

° *P = 0.003 (G1 vs. G3)*,

• *P = 0.036 (G2 vs. G3)*,

# *P = 0.01 (G1 vs. G3)*.

There was significantly higher baseline CD8 counts in G3 than the other two groups. Notably, mean CD8 counts were consistently above 1,000 cells/μL in G3 during the following 5 years of follow-up check (Figure 2A).

### Dynamics of Viral Reservoir During ART

To examine the viral reservoir decay dynamics of the three groups, we assessed levels of cell-associated HIV DNA (17, 18), HIV specific antibodies (19, 20), and PD-1 expression on CD4 T-cells (21–24) in this study.

**Figure 3:**
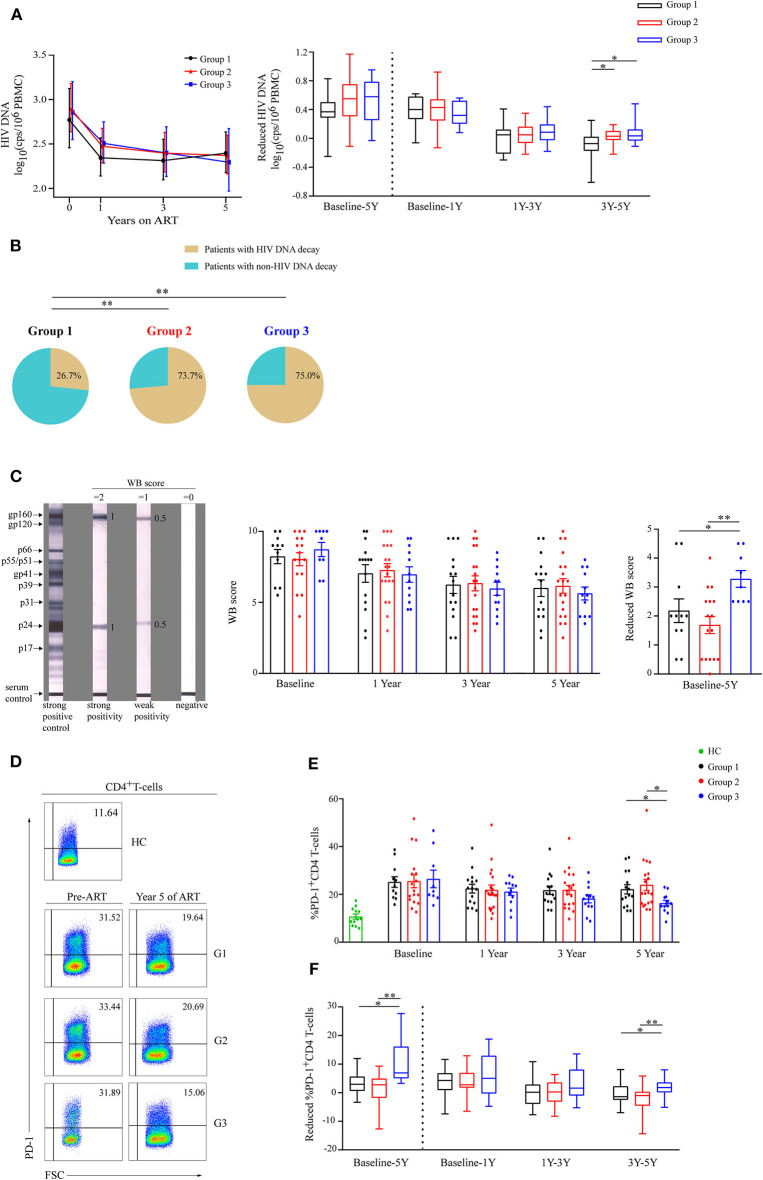
Analysis of viral decay among the three groups during ART. **(A)** Dynamics of HIV DNA are shown as geometric means (95% CI) among three groups during ART (left panel). Comparisons of reduced HIV DNA levels in three groups from baseline to year 5, from baseline to year 1, from years 1 to 3, and from years 3 to 5 (right panel). **(B)** The percentages of patients with reduced HIV DNA during years 3–5 of ART in the three groups. **(C)** Typical Western blot (WB) forms of strong positive control, strong reactive of gp160 and p24, weak reactive of gp160 and p24, and negative response are shown (left panel). Statistical analysis of WB score during ART (middle panel) and WB score decrease from baseline to year 5 (right panel) in three groups. **(D)** Representative flow cytometric data of PD-1 staining gated on CD4 T-cells. **(E)** Comparisons of the percentages of PD-1 expressions on CD4 T-cells in three groups during ART. **(F)** Changes in percentage of PD-1 expression on CD4 T-cells were compared in three different groups from baseline to year 5, from baseline to year 1, from years 1 to 3, and from years 3 to 5 (right panel). Group 1 (G1, *n* = 15 participants, 4 missing data at baseline), Group 2 (G2, *n* = 19 participants, 2 missing data at baseline), Group 3 (G3, *n* = 12 participants, 3 missing data at baseline), HC (health controls, *n* = 12 participants). In **(C,E)**, data are mean ± SEM. In (right panel of **A,F**), boxes show median and IQR, and whiskers are minimum and maximum. *P < 0.05, **P < 0.01. CI, confidence interval; SEM, standard error of the mean; cps, copies.

Cell-associated HIV DNA level is commonly used to quantitatively assess the HIV reservoir (17, 18). The levels of cell-associated HIV DNA decayed rapidly in the three groups within the first year of ART initiation (Figure 3A). At year 5 of ART, the level of HIV DNA tended to be lowest in G3 (Figure 3A), and a significantly reduced HIV DNA level only occurred in G3 after 3 years of ART (Figure 3A). In addition, the decay slope of HIV DNA was significantly higher in G3 compared to G1 during years 3–5 of ART (*P* = 0.01, Table 2). The percentages of patients with reduced HIV DNA between years 3 and 5 of ART were 26.7% (4/15), 73.7% (14/19), and 75% (9/12) in G1, G2, and G3, respectively (Figure 3B).

HIV specific antibodies could also be used to estimate viral reservoir size (19, 20). Based on the WB score (Figure 3C, left panel), we observed the dynamics of WB score in the three groups during ART (Figure 3C, middle panel), and WB score decreased most in G3 compared with the other groups from baseline to year 5 of ART (*P* < 0.05 and *P* < 0.01, respectively, Figure 3C, right panel).

As PD-1^+^CD4 T-cells is a preferential reservoir and the percentage of PD-1 expression on CD4 T-cells is positively correlated with the HIV reservoir (21–24), we also investigated PD-1 expression on CD4 T-cells (Figure 3D). We observed that the mean percentage of PD-1^+^CD4 T-cells was lowest in group 3 at year 5 of ART (Figure 3E), and the percentages of PD-1^+^CD4 T-cells significantly dropped in G3 particularly during years 3–5 of ART (Figure 3F).

These data suggest that G3 patients showed the most significant decrease in viral reservoir during 5 years of ART, especially in years 3–5 of ART. Therefore, patients with high CD8 counts may experience sustained viral reservoir decay in immune restored patients.

### Ki67 and PD-1 Expression on CD8 T-Cells During ART

**Figure 4:**
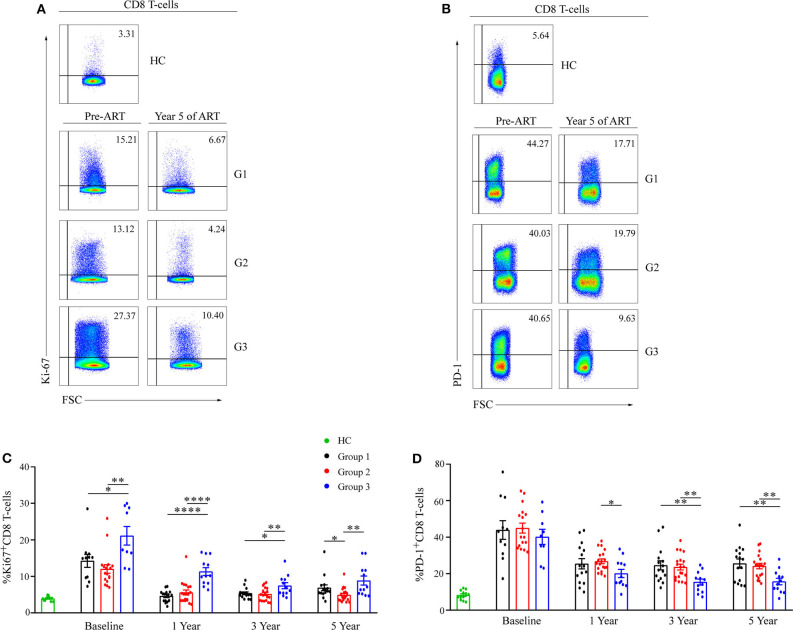
Ki67 and PD-1 expression on CD8 T-cell during ART. Representative flow cytometric data of Ki67 **(A)** and PD-1 **(B)** staining gated on CD8 T-cells. Comparisons of mean (SEM) of the percentages of Ki67 **(C)** and PD-1 **(D)** expressions on CD8 T-cells between groups during ART. Group 1 (G1, *n* = 15 participants, 4 missing data at baseline), Group 2 (G2, *n* = 19 participants, 2 missing data at baseline), Group 3 (G3, *n* = 12 participants, 3 missing data at baseline), HC (health controls, *n* = 12 participants). *P < 0.05, **P < 0.01, ****P < 0.0001.

To determine the mechanism of CD8 counts elevation, the expressions of Ki67 and PD-1 on CD8 T-cells were measured to evaluate cellular proliferation and exhaustion status, respectively. After initiation of ART, both Ki67 and PD-1 expression on CD8 T-cells dropped significantly among three groups during the first year (Figures 4A–D). The percentages of Ki67^+^CD8 T-cells maintained the highest levels in G3 before and after ART (Figure 4C). There were no differences in PD-1 expression on CD8 T-cells between groups at baseline; however, the mean percentage of PD-1^+^CD8 T-cells continued to be lowest in G3 after 1 years of ART (Figure 4D).

We also evaluated CD8 T-cell subsets (Supplementary Figure 2), HLA-DR, and CD38 co-expression on CD8 T-cells (Supplementary Figure 3) during ART. We found that HLA-DR and CD38 co-expression on CD8 T-cells was higher in G3 than the other groups during ART.

This data set indicates CD8 T-cells with a higher cellular proliferation activity and a lower immune exhaustion in G3 during long-term ART, and this may be the reason for a sustained high-level of CD8 T-cells in G3.

### Total and HIV-Specific CD8 Responses During ART

**Figure 5:**
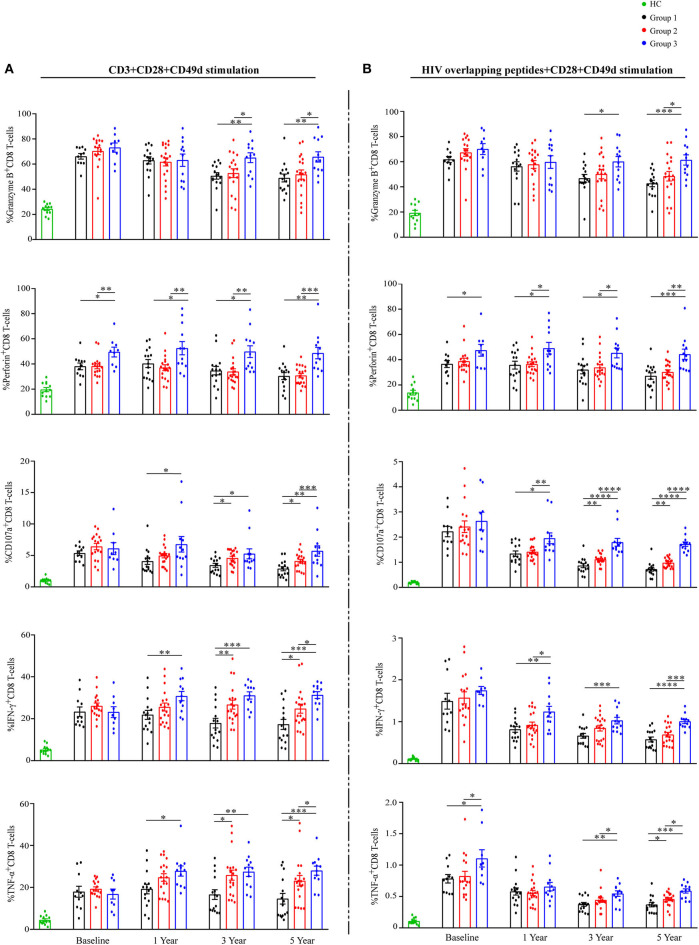
Total and HIV-specific CD8 T-cell functionalities during ART. Proportions of granzyme B, perforin, CD107a, IFN-γ, and TNF-α expression on CD8 T-cells **(A)** after stimulation with anti-CD3, and percentages of granzyme B, perforin, CD107a, IFN-γ and TNF-α expression on CD8 T-cells **(B)** after stimulation with HIV overlapping peptides show as mean ± SEM. Statistical analysis of functional markers mentioned above in three groups during ART, respectively. Group 1 (G1, *n* = 15 participants, 4 missing data at baseline), Group 2 (G2, *n* = 19 participants, 2 missing data at baseline), Group 3 (G3, *n* = 12 participants, 3 missing data at baseline), HC (health controls, *n* = 12 participants). *P < 0.05, **P < 0.01, ***P < 0.001, ****P < 0.0001.

To assess total CD8 T-cells functionalities, PBMCs were stimulated with anti-CD3 and analyzed for granzyme-B, perforin, CD107a, IFN-γ, and TNF-α expression on CD8 T-cells (Figure 5A and Supplementary Figure 4). No differences in any functional marker between groups at baseline were observed, with the following exception: the mean percentage of perforin^+^CD8 T-cells was higher in G3 than that in G1 and G2 (Figure 5A). At years 3 and 5 of ART, the proportions of each functional marker expression on CD8 T-cells were highest in G3, compared with the other groups (Figure 5A).

At the same time, PBMCs were stimulated with HIV overlapping peptides to assess HIV-specific CD8 T-cells responses (Figure 5B and Supplementary Figure 5). The proportions of CD8 T-cells producing CD107a, IFN-γ, and TNF-α decreased markedly in all groups during the first year of ART (Figure 5B). Similar to total CD8 T-cells functions reported above, the proportions of each functional marker expression on HIV-responsive CD8 T-cells were highest in G3 at years 3 and 5 of ART (Figure 5B). Taken together, CD8 T-cells in G3 have significantly higher functional capacity when compared with the other two groups.

### Correlation of Reduced HIV DNA and Increased CD4 Counts During Years 3–5 of ART With CD8 Function and Count

Finally, correlation analyses were conducted between reduced HIV DNA, increased CD4 counts, and CD8 counts including functional analysis during ART in all patients. Table 3A shows that reduced HIV DNA levels during years 3–5 were positively correlated with HIV-specific CD8 T-cells responses, including TNF-α expression level at year 5 (*r* = 0.294, *P* = 0.048), CD107a expression levels at years 3 and 5 (*r* = 0.476, *P* = 0.001; *r* = 0.375, *P* = 0.010, respectively). Participants with higher CD8 counts at years 1, 3, and 5 of ART had more reduced HIV DNA levels during years 3–5 of ART (*r* = 0.350, *P* = 0.017; *r* = 0.294, *P* = 0.047; *r* = 0.393, *P* = 0.007).

**Table 3:** (A) Reduced HIV DNA levels during years 3–5 of ART correlated to levels of CD8 function and counts over the course of ART.

**(A) Spearman correlation (*p*-value) with reduced HIV DNA levels**	**Baseline**	**1-year**	**3-year**	**5-year**
%IFN-γ^+^ HIV-specific CD8 T-cells	0.137 (0.419)	0.152 (0.313)	0.216 (0.150)	0.196 (0.191)
%TNF-α^+^ HIV-specific CD8 T-cells	0.049 (0.771)	0.159 (0.292)	0.288 (0.052)	0.294 (0.048)
%CD107a^+^ HIV-specific CD8 T-cells	0.017 (0.921)	0.049 (0.747)	0.476 (0.001)	0.375 (0.010)
%Granzyme B^+^CD8 T-cells	0.103 (0.544)	−0.212 (0.157)	0.033 (0.829)	0.116 (0.443)
%Perforin^+^CD8 T-cells	−0.040 (0.816)	−0.189 (0.208)	−0.136 (0.369)	0.168 (0.263)
CD8 count (cells/μl)	0.190 (0.205)	0.350 (0.017)	0.294 (0.047)	0.393 (0.007)
**(B) Spearman correlation (*****p*****-value) with increased CD4 counts**	**Baseline**	**1-year**	**3-year**	**5-year**
%IFN-γ^+^ HIV-specific CD8 T-cells	0.340 (0.039)	0.239 (0.110)	0.318 (0.031)	0.371 (0.011)
%TNF-α^+^ HIV-specific CD8 T-cells	0.258 (0.124)	0.235 (0.116)	0.326 (0.027)	0.288 (0.052)
%CD107a^+^ HIV-specific CD8 T-cells	0.249 (0.137)	0.329 (0.026)	0.322 (0.029)	0.297 (0.045)
%Granzyme B^+^CD8 T-cells	0.094 (0.579)	0.228 (0.127)	0.287 (0.053)	0.351 (0.017)
%Perforin^+^CD8 T-cells	0.267 (0.110)	0.385 (0.008)	0.284 (0.056)	0.314 (0.034)
CD8 count (cells/μl)	−0.101 (0.506)	0.120 (0.427)	0.064 (0.674)	0.301 (0.042)

*(B) Increased CD4 counts during years 3–5 of ART correlated to levels of CD8 function and counts over the course of ART. IFN-γ, interferon-γ; TNF-α, tumor necrosis factor-α*.

As shown in Table 3B, increased CD4 counts during years 3–5 were positively correlated with CD8 functions between years 3–5 of ART as follows: IFN-γ expression level at years 3 and 5 (*r* = 0.318, *P* = 0.031; *r* = 0.371, *P* = 0.011, respectively), TNF-α expression level at year 3 (*r* = 0.326, *P* = 0.027), CD107a expression levels at years 3 and 5 (*r* = 0.322, *P* = 0.029; *r* = 0.297, *P* = 0.045, respectively), granzyme B expression levels at year 5 (*r* = 0.351, *P* = 0.017), and perforin expression levels at year 5 (*r* = 0.314, *P* = 0.034). However, similar associations were only observed between increased CD4 counts during years 3–5 and CD8 counts at year 5 of ART (*r* = 0.301, *P* = 0.042).

## Discussion

The aim of this study is to investigate the effect of different levels of CD8 T-cells on CD4 recovery and viral reservoir decay under the condition of CD4 recovery above 500 cells/μL after 5 years of ART. We found that both CD4 counts increased and viral reservoir decay were highest in the persistently high CD8 counts group (G3) compared to the other groups during 5 years of ART. Rate of CD4 counts gain and rate of HIV DNA levels decay were rapid within the first year of ART in all the three groups. These results were consistent with previous reports that CD4 recovery and HIV DNA decline occurred mainly in the first 2 years of ART (16, 25). This may be the result of productively infected cell death and prevention of new cell infections (26).

Our results also showed that an increase in CD4 counts in the first year of ART was significantly lower in G3 than that in the other two groups. This result was consistent with the report that elevated CD8 counts at ART initiation associated with poor CD4 recovery (1). However, within 5 years of ART especially during years 3–5 of ART, the patients in G3 had the most significant increase in CD4 counts. This result is different from the report that high CD8 levels were associated with poor CD4 recovery during long-term ART (1, 6), which may be due to the fact that this study enrolled patients without any CD4 level restrictions during 2 years of ART. We also found elevated CD8 counts associated with poor CD4 increase in patients with poor CD4 reconstitution within 3 years of ART (unpublished date). In addition, a previous study has shown that a higher CD4 counts was associated with some slight benefit for HIV-infected individuals with CD4 counts above 500 cells/μL (27). We presumed that a higher CD4 count in G3 may be more beneficial for immune restored patients.

We found that HIV DNA was significantly reduced in G3 patients during years 3–5 of ART which was different from G1 and G2. More importantly, we also found that the HIV reservoir size decreased significantly in G3 and tended to be the smallest in G3 compared with the other groups after 5 years of ART. Therefore, this study provides evidence that persistent viral reservoir decay is likely to occur in immune restored patients with higher CD8 counts after long-term ART. Viard et al. reported that HIV DNA levels did not significantly decrease after 3 years of ART (28); however, other studies reported that some individuals had continuous viral reservoir decay after long-term ART (29). Chun et al. reported that the HIV-1 proviral DNA load in CD4 T-cells showed no correlation with CD8 counts in treated patients, but an inverse correlation with CD4:CD8 ratio (30). In our study, patients with CD4 recovery above 500 cells/μL after 5 years of ART were enrolled, which was different from previous studies. Long-term non-progressors with low HIV reservoir have high levels of CD8 counts (13), which suggests that patients in G3 maybe have a similar immune status. Our results suggest high CD8 counts are conducive to viral decay in patients with immune restoration during long-term ART. It may explain why individuals with high CD8 counts have a reduced risk of AIDS-related events during ART (1). Nevertheless, Trickey et al. found that high CD8 counts were associated with AIDS mortality in this population with high CD4 counts and suppressed viral loads (14). The above-mentioned opposite outcomes may be attributed to discrepancies in study subjects.

Owing to the HIV reservoir, it has been estimated that achieving a “sterilizing” cure would need more than 60 years of ART alone (31). The smaller the reservoir size, the longer time the virus would need in order to rebound (32). Whether immune restored patients with high CD8 counts are more likely to accelerate decay of the HIV reservoir and achieve a functional cure requires further investigation.

In order to explore the mechanism further, we compared the dynamic changes of CD8 T-cells function in the three groups. High PD-1 expression on CD8 T-cells were linked to impaired proliferation and dysfunction (33) and associated with HIV persistence (34), and Ki67 is a common marker for proliferation. Consistent with previous reports (35), our results illustrated that HIV-specific CD8 cytotoxic T-cells (CTL), the expression of PD-1, and ki67 on CD8 T-cells all decreased rapidly during the first year of ART. However, after the first year of ART, G3 patients had remarkably higher frequencies of functional CD8 T-cells than the other two groups. The expression of PD-1 on CD8 T-cells maintained at the lowest levels in G3 after 1 year of ART. As blocking PD-1 could reverse the CTL response (36), the persistence low levels of PD-1 on CD8 T-cells coincided with the most potent CD8 T-cell function in G3 after 1 year of ART.

As increased CD4 counts and reduced viral levels during years 3–5 of ART were both higher in G3 than the other groups, we then analyzed the relationships between the above-mentioned two indictors and CD8 counts and function. We found that increased CD4 counts and reduced viral levels were associated with CD8 counts and function at years 3 and 5 of ART. These results are consistent with the report that HIV-specific CD8 cytotoxic T-cells (CTL) (37) and strong CD8 T-cells responses (35, 38) were beneficial to HIV reservoir decay. Dysfunctional CD4 T-cells could contribute to the exhaustion of the CD8 T-cells (39), and in this study we enrolled patients with CD4 recovery above 500 cells/μL after long-term ART. Therefore, whether CD4 T-cell immune recovery helps restoration of CD8 T-cells function is worth further study. Inherent characteristics may play an important role between the three groups (Supplementary Figure 6).

The mechanisms of sustained CD8 elevation remain largely unclear, which may include CMV co-infection (40, 41), bystander activation (42), and a shifted compartment distribution from lymphoid tissues (8). In addition, there are some limitations to this study. Firstly, as male-to-male sex is the main route of HIV transmission in major cities of China (43) at present, most HIV-infected individuals in our center are men. Participants were overwhelmingly male in this study, thus the results do not represent the situation of HIV-infected women. Secondly, the latent reservoir is mainly located in lymphoid tissues rather than in the peripheral blood (44, 45). Unfortunately, our study only obtained peripheral blood samples, without corresponding lymphoid tissues. Besides, patients with longer-term (e.g., >5-years) effective ART were not enrolled in our study. The decay slopes of the latent reservoir are variant among patients in different term of ART, which is greatest within 5 years after initiation of ART. This decay tends to be stable thereafter (29). Finally, the case number was small, and studies with a larger sample size and a longer follow-up period would be better to make sound conclusions.

In conclusion, our findings showed that CD4 recovery and viral reservoir decay were associated with persistently high CD8 counts in immune restored patients during long-term ART. The information provided herein will contribute to a better understanding of CD8 persistence on viral reservoir decay and immune recovery under ART.

## Data Availability Statement

The datasets generated for this study are available on request to the corresponding author.

## Ethics Statement

The studies involving human participants were reviewed and approved by the institutional review boards of Fifth Medical Center of Chinese PLA General Hospital. The patients/participants provided their written informed consent in line with the Declaration of Helsinki to participate in this study.

## Author Contributions

L-XZ, Y-MJ, CZ, J-WS, and F-SW conceived the study, designed the experiments, and analyzed the data. L-XZ and Y-MJ performed the experiments. XF, R-NX, H-HH, J-YZ, L-FW, C-BZ, LJ, and MS contributed to reagents and materials. L-XZ, Y-MJ, and F-SW wrote the article. All authors read and approved the final manuscript.

## Conflict of Interest

The authors declare that the research was conducted in the absence of any commercial or financial relationships that could be construed as a potential conflict of interest.
