# Supplementary material for: HIV Reservoir Decay and CD4 Recovery Associated With High CD8 Counts in Immune Restored Patients on Long-Term ART
Source: Front Immunol. 2020 Jul 23;11:1541. doi: 10.3389/fimmu.2020.01541 (PMC7390854; doi:10.3389/fimmu.2020.01541)

Supplementary Table 1. Characteristics of patients with serial samples in the three groups analyzed

	Group 1 (N=15)	Group 2 (N=19)	Group 3 (N=12)	*P*
Male	14 (93.3)	19 (100)	11 (91.7)	0.504
Mode of transmission				
Male-to-male sex	6 (40.0)	14 (73.7)	9 (75.0)	0.093
Heterosexual	6 (40.0)	3 (15.8)	3 (25.0)	0.280
Undetermined	3 (20.2)	2 (10.5)	0	0.300
Age at ART initiation (years)	43 (37-50)	33 (26-46)	28 (26-48)	0.084
Pre-ART plasma HIV RNA (log_{10} copies/mL)	4.10 (3.40-5.10)	4.15 (3.35-5.30)	4.17 (3.38-5.80)	0.185
Pre-ART CD4 T-cell count (cells/μL)	345 (274-477)	366 (254-524)	338 (262-483)	0.817
Pre-ART CD8 T-cell count (cells/μL)	720 (593-923) △	1157 (781-1518) ▲	1563 (852-1943) △▲	0.003
ART regimens at initial stage				
2 NRTIs + 1 NNRTIs	15 (100)	18 (94.7)	12 (100)	1.000
2 NRTIs + 1 PIs	0	1 (5.3)	0	1.000

Data are n (%) or median (IQR). Abbreviations: ART, antiretroviral therapy; HIV, human immunodeficiency virus; IQR, interquartile range; NRTIs, nucleoside reverse transcriptase inhibitors; NNRTIs, non- nucleoside reverse transcriptase inhibitors; PIs, protease inhibitor.

△ *P* =0.001 (G1 vs G3), ▲*P* =0.030 (G2 vs G3).

Supplementary Figure 1. Distribution of 203 chronic HIV infected patients according to different levels of CD4 and CD8 counts after 5 years of ART

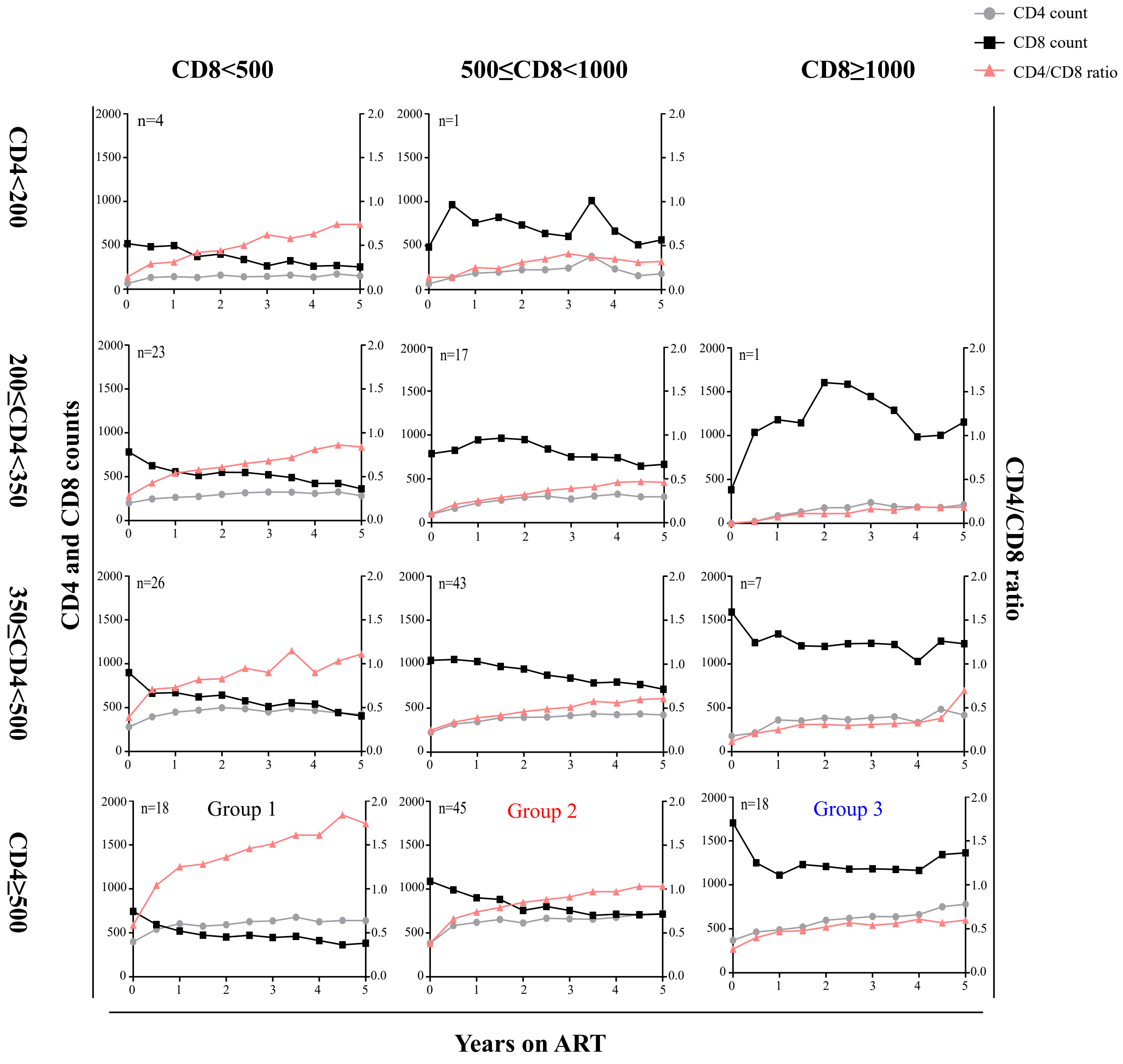

Supplement Figure 2. Changes of CD8 T-cell subsets during ART

(A) Gating strategy of CD8 T-cell subsets. Naive (N): CD45RA+CCR7+; Central memory (CM): CD45RA-CCR7+; Effector memory (EM): CD45RA-CCR7-; CD45RA-expressing effector memory cells (EMRA): CD45RA+CCR7-. Comparisons of mean (SEM) of the percentages of T_{N} (B), T_{CM} (C), T_{EM} (D) and T_{EMRA} (E) CD8 T-cells in the three groups during ART, respectively. Group 1 (n=15 participants, 4 missing data at baseline), Group 2 (n=19 participants, 2 missing data at baseline), Group 3 (n=12 participants, 3 missing data at baseline), HC (health controls, n=12 participants). *P <0.05, **P <0.01.

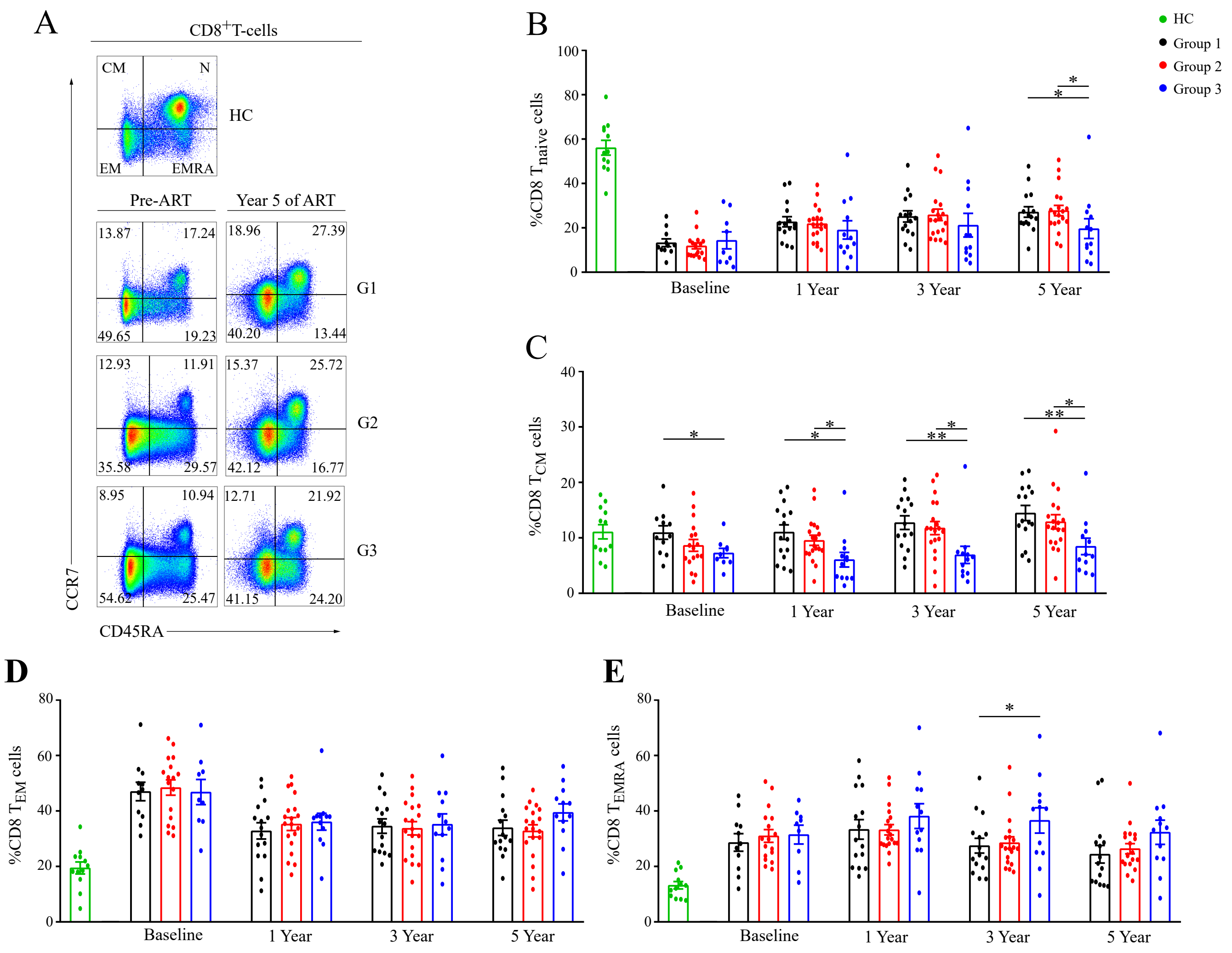

Supplement Figure 3. CD38 and HLA-DR co-expression on CD8 T-cells during ART

(A) Representative flow cytometric data of CD38 and HLA-DR co-expression staining gated on CD8 T-cells.
(B) Statistical analysis of CD38 and HLA-DR co-expression on CD8 T-cells in three groups during ART.
Group 1 (n=15 participants, 4 missing data at baseline), Group 2 (n=19 participants, 2 missing data at baseline), Group 3 (n=12 participants, 3 missing data at baseline), HC (health controls, n=12 participants). *P <0.05, **P <0.01.

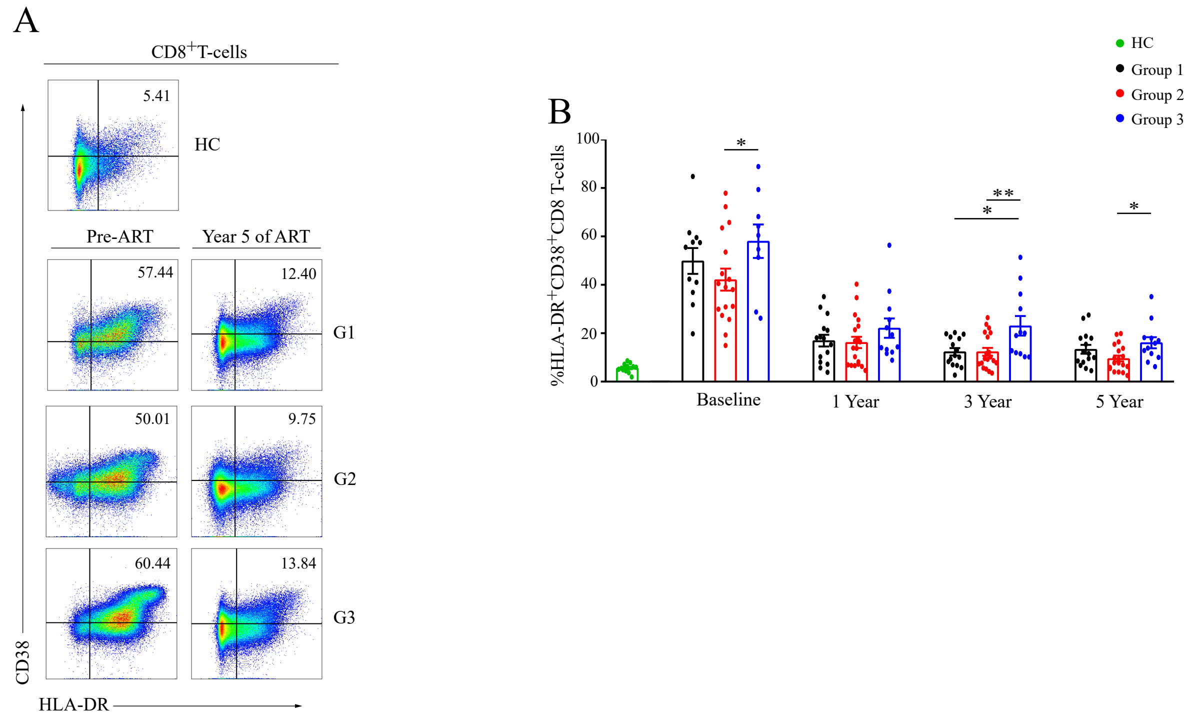

Supplement Figure 4. Gating strategy for total CD8 T-cell responses

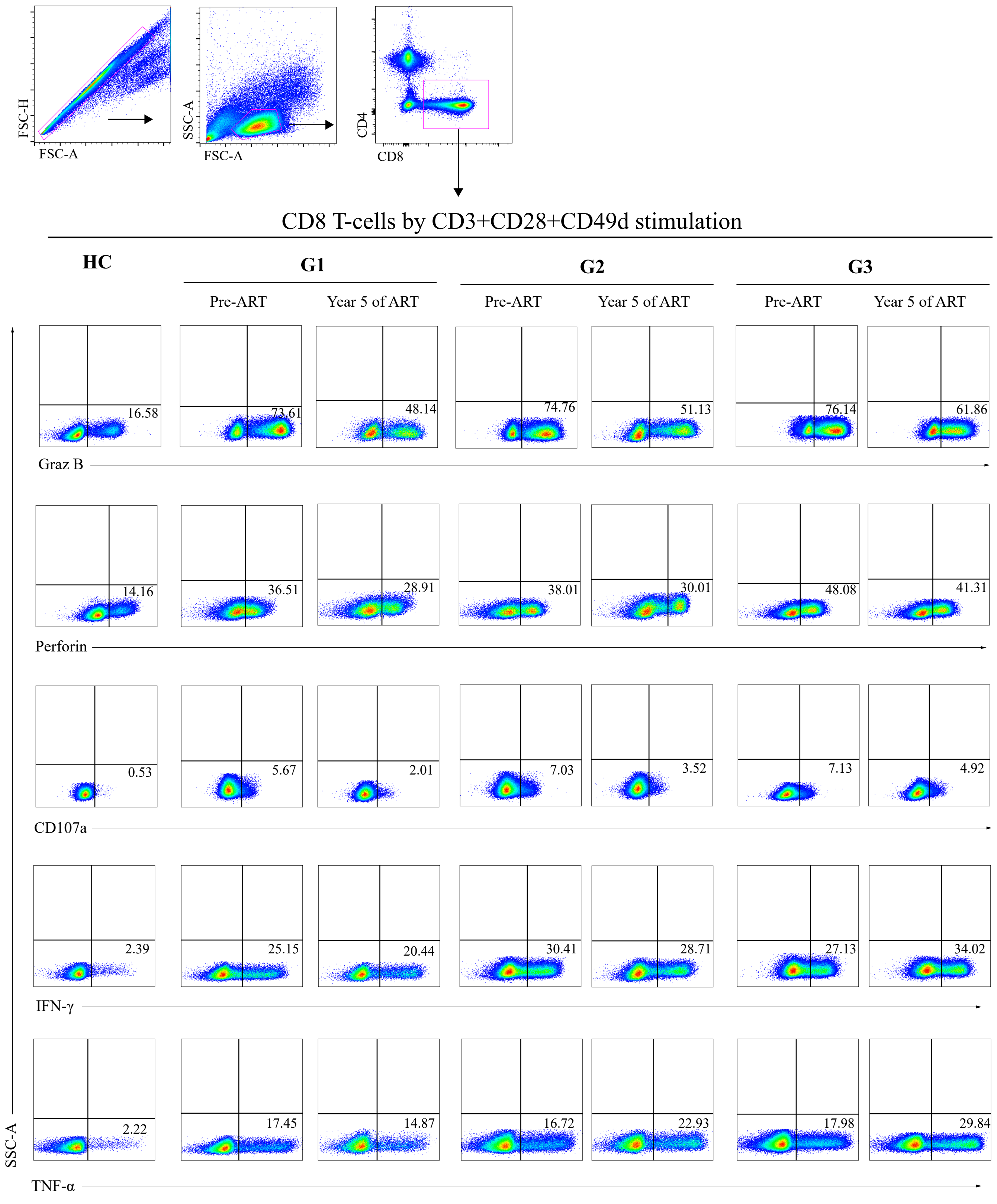

Supplement Figure 5. Gating strategy for HIV-specific CD8 T-cell responses

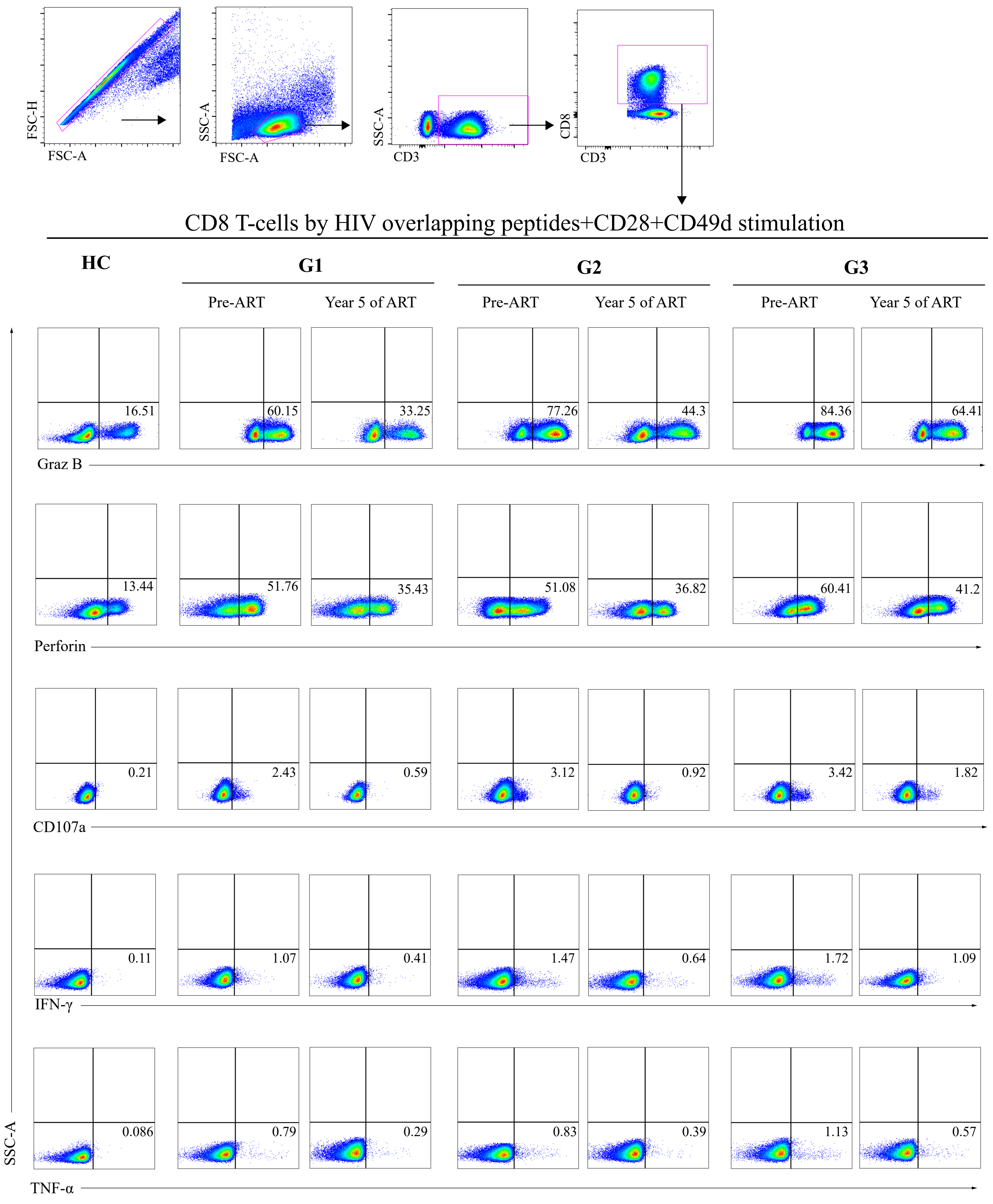

Supplementary Figure 6. Distribution of HLA-B allele among three groups

Protective and risk allele frequencies of HLA-B are shown in Group 1 (A), Group 2 (B) and Group 3 (C), respectively. (D) Distribution of protective and risk allele frequencies of HLA-B in the three groups. Group 1 (n=15 participants), Group 2 (n=19 participants), Group 3 (n=12 participants). ***P* <0.01.

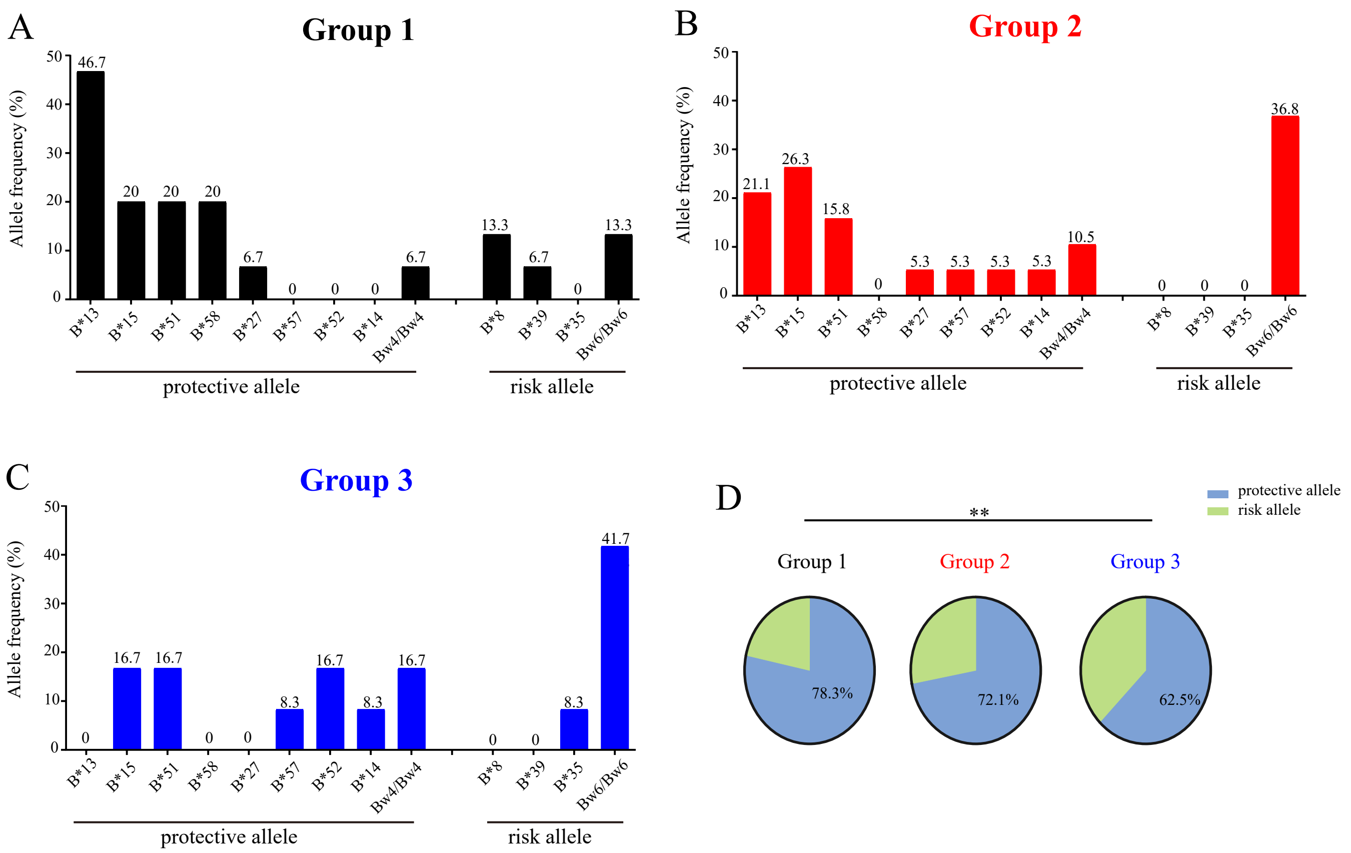

Supplement: Supplementary file 1 [file Data_Sheet_1.pdf]
