# Supplementary material for: HIV Reservoir Decay and CD4 Recovery Associated With High CD8 Counts in Immune Restored Patients on Long-Term ART
Source: Front Immunol. 2020 Jul 23;11:1541. doi: 10.3389/fimmu.2020.01541 (PMC7390854; doi:10.3389/fimmu.2020.01541)

Appendix Figure 1. Changes of IP-10 level during ART

(A) Statistical analysis of IP-10 level in three groups during ART. Group 1 (n=15 participants, 4 missing data at baseline), Group 2 (n=19 participants, 2 missing data at baseline), Group 3 (n=12 participants, 3 missing data at baseline), HC (health controls, n=12 participants). **P* <0.05.

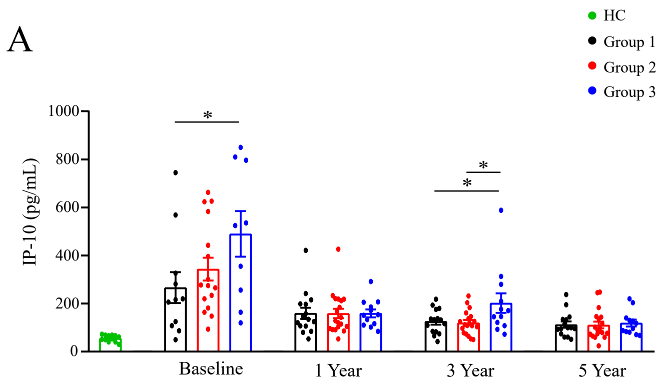

Appendix Figure 2. The relationship between PD-1 expression on CD8 T-cells and CD8 T-cell activation

PD-1 expression on CD8 T-cells is associated with CD8 T-cell activation in all participants (A, n=175), in Group 1 (B, n=56), in Group 2 (C, n=74) and in Group 3 (D, n=45) during 5 years of ART (including at baseline, years 1, 3 and 5), respectively.

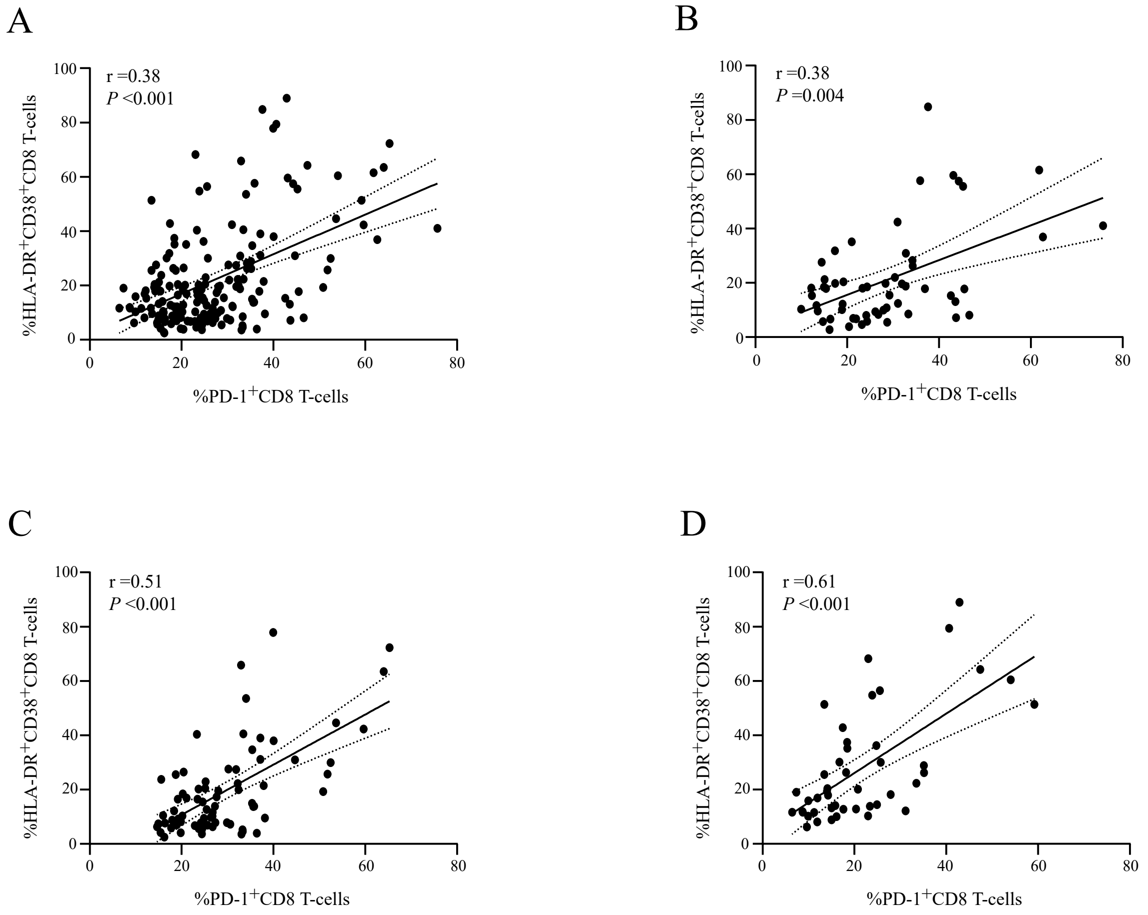

Supplement: Supplementary file 2 [file Data_Sheet_2.PDF]
